# Metabolic Analysis and Renal Protective Effects of Linagliptin and Empagliflozin in Alport Syndrome

**DOI:** 10.34067/KID.0000000000000472

**Published:** 2024-05-23

**Authors:** Mengyuan Ge, Judith Molina, Ian Tamayo, Guanshi Zhang, Jin-Ju Kim, Rachel Njeim, Flavia Fontanesi, Michael Paul Pieper, Sandra Merscher, Kumar Sharma, Alessia Fornoni

**Affiliations:** 1 Katz Family Division of Nephrology and Hypertension, Department of Medicine, University of Miami Miller School of Medicine, Miami, Florida; 2 Peggy and Harold Katz Family Drug Discovery Center, University of Miami Miller School of Medicine, Miami, Florida; 3 Center for Precision Medicine, School of Medicine, University of Texas Health San Antonio, San Antonio, Texas; 4 Department of Biochemistry and Molecular Biology, University of Miami, Miami, Florida; 5 Cardiometabolic Diseases Research, Boehringer Ingelheim Pharma GmbH & Co. KG, Biberach, Germany

**Keywords:** Alport syndrome, DPP4 inhibitor, SGLT2 inhibitor, metabolites

## Abstract

**Key Points:**

- Linagliptin reduces kidney function decline and extends lifespan in Alport syndrome mice.
- Inhibiting the generation of glucose metabolites could serve as a potential therapeutic strategy for the treatment of Alport syndrome.

**Background:**

We previously demonstrated that empagliflozin (Empa), a sodium-glucose cotransporter-2 inhibitor, reduces intrarenal lipid accumulation and slows kidney function decline in experimental Alport syndrome (AS). In this study, we aimed to evaluate the renal protective benefits of linagliptin (Lina), a dipeptidyl peptidase-4 inhibitor in AS, and compare it with Empa.

**Methods:**

Metabolite distribution in kidney cortices was assessed using mass spectrometry imaging. We examined albuminuria and histological changes in kidneys from AS mice treated with Lina and/or Empa or vehicle.

**Results:**

Several metabolites, including adrenic acid and glucose, were increased in renal cortices of AS mice compared with wild-type (WT) mice, whereas eicosapentaenoic acid levels were decreased. In addition, a redistribution of adrenic acid from the glomerular compartment in WT mice to the tubulointerstitial compartment in AS mice was observed. Both Lina and Empa treatments were found to reduce albuminuria to extend the survival of AS mice for about 10 days and to decrease glomerulosclerosis and tubulointerstitial fibrosis compared with WT mice. There were no significant differences with regard to the renal phenotype observed between Empa- and Lina-treated AS mice, and the combination of Lina and Empa was not superior to individual treatments. *In vitro* experiments revealed that dipeptidyl peptidase-4 is expressed in podocytes and tubular cells derived from both AS and WT mice. Differently from what we have reported for Empa, Lina treatment was found to reduce glucose-driven respiration in AS tubular cells but not in AS podocytes.

**Conclusions:**

Renal expression patterns and spatial distribution of several metabolites differ in AS compared with WT mice. Although Lina and Empa treatments similarly partially slow the progression of kidney disease in AS, the metabolic mechanisms conferring the protective effect may be different.

## Visual Abstract

**Figure:**
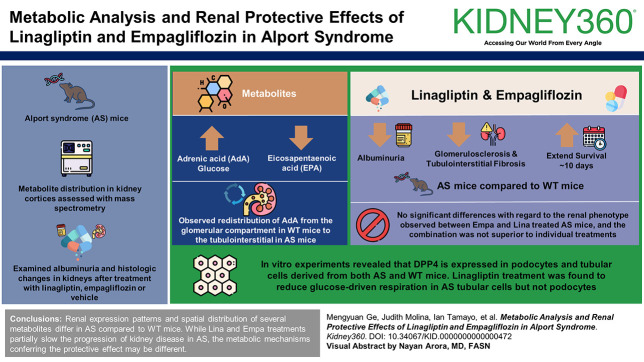

## Introduction

Dipeptidyl peptidase-4 (DPP4) inhibitors and sodium-glucose cotransporter-2 (SGLT2) inhibitors are both second-line options for managing glucose levels in patients with type 2 diabetes.^1,2^ More recently, SGLT2 inhibitors (SGLT2i) were found to protect from CKD progression even in the absence of diabetes.^3,4^ SGLT2i reduce the reabsorption of glucose in the proximal tubules, thereby reducing blood glucose levels.^5^ Conversely, DPP4 inhibitors (DPP4i) block the DPP4 enzyme, which prevents the inactivation of glucagon-like peptide 1 (GLP-1) and stimulates insulin secretion.^6,7^ Both treatments lead to a decrease in glucose availability as a metabolic substrate, potentially causing adaptations in substrate utilization. Alport syndrome (AS) is a genetic condition first identified as hereditary familial congenital hemorrhagic nephritis.^8^ Characterized by mutations in collagen type 4 genes (*COL4A3*, *COL4A4*, *COL4A5*),^9,10^ AS results in progressive kidney issues and abnormalities in both the inner ear and the eye.^11,12^ Although AS is known as a nonmetabolic CKD, studies have shown disruptions in lipid metabolism in AS.^13–15^ We have demonstrated that increased triglyceride and esterified cholesterol content, along with free fatty acid uptake, contributes to the progression of AS.^13–16^ Although there are currently no preventive or curative therapies for AS, research suggests that reducing intrarenal lipid accumulation, such as by promoting the utilization and elimination of accumulated lipids, may offer a therapeutic option to prevent the decline in kidney function in AS.^13–15,17,18^ We previously demonstrated that SGLT2i by empagliflozin prevents kidney function decline in experimental AS by lowering intrarenal lipid accumulation.^19^ In this process, SGLT2 inhibition reduces glucose reabsorption and promotes the utilization of lipids as an energy source in podocytes. As a glucose-lowering treatment for type 2 diabetes, DPP4is may cause a similar effect in substrate switch. In support, DPP4i were found to effectively lower cholesterol levels in LDL receptor-deficient mice.^20^ A systematic literature review also indicated that DPP4i is associated with a significant reduction in total cholesterol in patients with type 1 or type 2 diabetes.^21^ Moreover, it has been reported that DPP4i treatment increases glucose and fatty acid uptake in the hypertrophied heart of nondiabetic mice, leading to improved myocardial energy metabolism.^22^ In this study, we investigated the metabolic mechanisms involved in the renoprotective benefits of linagliptin (Lina) in a mouse model of AS (*Col4a3*^−/−^ mice). In addition, we compared the Lina-mediated effects to the effect mediated by empagliflozin (Empa) in AS mice.

## Methods

### Animal Studies

#### Study Approval

All studies involving mice were approved by the Institutional Animal Care and Use Committee at the University of Miami (UM). The UM has an Animal Welfare Assurance on file with the Office of Laboratory Animal Welfare, National Institutes of Health (A-3224-01, effective November 24, 2015). In addition, UM is registered with the US Department of Agriculture Animal and Plant Health Inspection Service, effective December 2014, registration 58-R-007. As of October 22, 2013, the Council on Accreditation of the Association for Assessment and Accreditation of Laboratory Animal Care International has continued UM's full accreditation.

#### Phenotypic Analysis of Mice

*Col4a3*^−/−^ mice (a mouse model of AS) are in a 129X1/SvJ background and were purchased from Jackson Laboratory (129-Col4a3tm1Dec/J, stock 002908). *Col4a3*^+/−^ littermates were bred to generate *Col4a3*^−/−^ mice, which were genotyped. Genotyping of mice was performed using DNA isolated from tail clipping samples. DNA was isolated and amplified using REDExtract-N-Amp Tissue PCR Kit (Sigma XNAT). The specific primers used were *Col4a3*KO (forward: GCTATCAGGACATAGCGTTGG) and wild type (WT) (forward: TGCTCTCTCAAATGCACCAG). The reverse sequence is identical for both WT and AS (reverse: CCAGGCTTAAAGGGAAATCC). The PCR products were analyzed on a 3% agarose gel. Expected results are mutant (AS)=230 bp, heterozygote=230 bp and 309 bp, and WT=309 bp. Mice were assigned to the individual study groups as described below. Four-week-old *Col4a3*^−/−^ (AS) mice were treated with the following doses of Lina and/or Empa in their chow: 40 mg/kg Lina and 70 mg/kg Empa. The following study groups were analyzed: WT, AS, AS+Lina, AS+Empa, and AS+Empa+Lina. Both male and female mice were included in the study. The mice were fed before being euthanized. The survival study was carried out in AS mice where treatment was initiated at 4 weeks of age. The purpose of this study was to see if Lina and Empa treatment of mice with AS may postpone ESKD and thereby prolong survival. Additional information of methods can be found in the Supplemental Material.

### Matrix-Assisted Laser Desorption/Ionization Mass Spectrometry Imaging for Spatial Metabolomics

Kidney samples for matrix-assisted laser desorption/ionization mass spectrometry imaging (MALDI-MSI) were prepared as previously described.^23^ Briefly, kidneys were snap frozen in liquid nitrogen and stored at −80°C until time of sectioning. Frozen tissues were serially sectioned using a cryo-stat with chamber temperature set to −20°C. Three serial kidney sections were analyzed per mouse (10 *µ*m). For spatial metabolomics analysis of mice kidney samples, the Q Exactive HF-X orbitrap mass spectrometer (Thermo) coupled to a ultraviolet-laser enabled MALDI source (Spectroglyph LLC) was used. We have optimized protocols for mice kidney samples, developed quality control/quality assurance, increased molecular coverage, improved metabolite spatial resolution, implemented optical visualization and data analysis workflows, and demonstrated the value of spatial metabolomics data to guide integrative efforts.^24,25^ Small metabolite analysis was performed using 1,5-diaminonaphthalene matrix at a sprayed concentration of 5.5 mg/ml dissolved in 50% ethanol, 5% 1.0 N HCl, and 45% H_2_O. The spray conditions included a flow rate of 25 *µ*l/min, 20 total passes, a nozzle temperature of 80°C, and a sheath pressure of 10 psi N_2_. MALDI acquisition parameters include Spectroglyph MALDI source, 20 *µ*m resolution, and 2.6 µJ laser energy; mass spectrometry acquisition parameters include Thermo QE HF-X Orbitrap, negative polarity, and 100–1000 m/z range. By localizing small molecules within specific glomerular and tubular compartments *via* autofluorescence, brightfield microscopy and PAS staining on the same and adjacent serial sections,^26–30^ we used the spatial metabolomics data to investigate metabolic reprogramming across microanatomical regions and cell types. For processing of MSI data, .raw and .xml files were imported into the Image Insight software and converted to .imzML and .ibd files. Data were then submitted to METASPACE web-based annotation platform for metabolite annotation and intensity extraction. Briefly, metabolite intensity values were extracted and averaged for each pixel within an image dataset for all annotated metabolites at 20% false discovery rate threshold. These raw data were uploaded to MetaboAnalyst v5.0 statistical software for comparative analysis between groups including a heat map of top metabolites, clustering by principal component analysis, partial least squares discrimination analysis, and variables of importance scoring (VIP) from partial least squares discrimination analysis.

### Cellular Respiration Measurements

To measure the oxygen consumption rate, a high-resolution respirometer (O2k-Fluo-Respirometer, Oroboros Instruments, Innsbruck, Austria) filled with 2 ml of mitochondrial respiration buffer (MiR05) was used. The buffer contained 0.5 mM EGTA, 3 mM MgCl_2_ 6H_2_O, 60 mM K-lactobionate, 20 mM taurine, 10 mM KH_2_PO_4_, 20 mM HEPES, 110 mM sucrose, and 1 g/L of fatty acid-free BSA, and the respirometer was set at 37°C. The Substrate-Uncoupler-Inhibitor-Titration-002 protocol was followed with some modifications as previously described.^19^ The cell respiration was measured as pmol O_2_ consumed per second and then normalized to the cell number.

### Statistical Analyses

For each statistical test, biological sample size (*n*) and *P* value are indicated in the corresponding figure legends. The results are presented as mean±SD. Statistical analysis was conducted using Prism GraphPad 7 software. To identify significant outliers, GraphPad's outlier calculator was employed, and any outliers found were excluded from further statistical analysis. The animals were categorized into groups on the basis of their genotypes and subsequently randomized, with the investigators blinded to the analysis. Two-tailed Student *t* test was used for comparing two groups while one-way ANOVA followed by the Holm-Sidak multiple comparison test was applied for other analyses. The survival curve analyses were performed using the log-rank Mantel-Cox test. A *P* value below 0.05 was considered statistically significant. Only data from independent experiments were analyzed.

## Results

### Differential Abundance and Spatial Distribution of Metabolites in Alport Mouse Kidney Cortices Compared with WT

**Figure 1:**
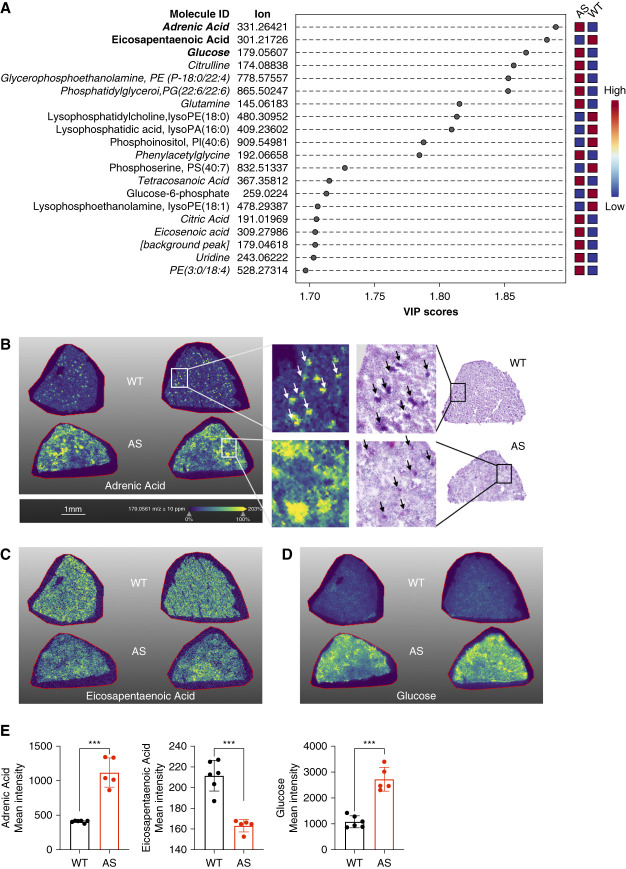
**MALDI-MSI analysis reveals differential accumulation and spatial distribution of metabolites in Alport mice**. (A) PLS-DA identifies key discriminant metabolites between AS and WT kidneys. *Italic*: metabolites that are upregulated in AS. (B) Molecular ion images of AdA and PAS staining on adjacent serial sections of WT and AS mouse kidneys, with white arrows indicating AdA distribution and black arrows indicating glomeruli. (C and D) Molecular ion images of EPA and glucose in WT and AS mouse kidneys. (E) Quantification of AdA, EPA, and glucose levels in WT and AS mouse kidneys. **P < 0.001. Two-tailed Student *t* test. AdA, adrenic acid; AS, Alport syndrome; EPA, eicosapentaenoic acid; MALDI-MSI, matrix-assisted laser desorption/ionization mass spectrometry imaging; PLS-DA, partial least squares discriminant analysis; WT, wild type.

Dysregulation of metabolites, such as neutral lipids, has been identified in both the glomerular and tubular sections of the kidneys in AS mice.^13,14,19^ These metabolic irregularities in AS arise from an excess of cholesterol influx,^13^ diminished utilization of fatty acids,^18^ and an elevation in fatty acid uptake.^14^ To investigate the specific metabolites that are dysregulated in AS kidneys compared with WT kidneys, we used a high-end mass spectrometry imaging setup MALDI-Q Exactive HF-X MSI (Thermo) for spatial metabolomics analysis. This technique can reveal metabolic reprogramming across microanatomical regions and cell types. The MALDI-MSI analysis of kidney cortex tissue regions in AS mice samples revealed alterations in several metabolites (Figure 1A and Supplemental Table 1). Specifically, we found reduced levels of eicosapentaenoic acid (EPA), lysophosphatidylcholine, lysophosphatidic acid, phosphatidylinositol, phosphoserine, glucose-6-phosphate, and lysophosphoethanolamine in AS kidneys, whereas adrenic acid (AdA), glucose, citrulline, glycerophosphoethanolamine, phosphatidylglycerol, glutamine, phenylacetylglycine, tetracosanoic acid, citric acid, eicosenoic acid, uridine, and phosphatidylethanolamine were found to accumulate (Figure 1, A–E). Similarly, principal component analysis showed that metabolites in AS and WT kidneys distinctly clustered together (Supplemental Figure 1). The top three metabolites that exhibited the most significant changes in AS were AdA, EPA, and glucose. AdA and EPA are omega-6 and omega-3 polyunsaturated fatty acids (PUFAs), respectively.^31,32^ It is noteworthy that AdA was found to be primarily localized in the glomerular region in WT kidneys, whereas in the AS kidney, AdA was found to be dispersed throughout the tubular area (Figure 1B). Unexpectedly, we found that glucose accumulated in the kidney sections of AS mice (Figure 1D), although AS is considered a kidney disease of nonmetabolic origin. Nevertheless, this observation may explain why SGLT2i were found to exhibit beneficial effects on renal disease progression in CKD patients without diabetes in the prevention of adverse outcomes in CKD trial and in the The Study of Heart and Kidney Protection with Empagliflozin trial,^3,4^ and it reinforces the viability of using SGLT2i as a treatment for with kidney diseases of nonmetabolic origin, including AS.

### Linagliptin Improves Renal Function in a Mouse Model of AS

**Figure 2:**
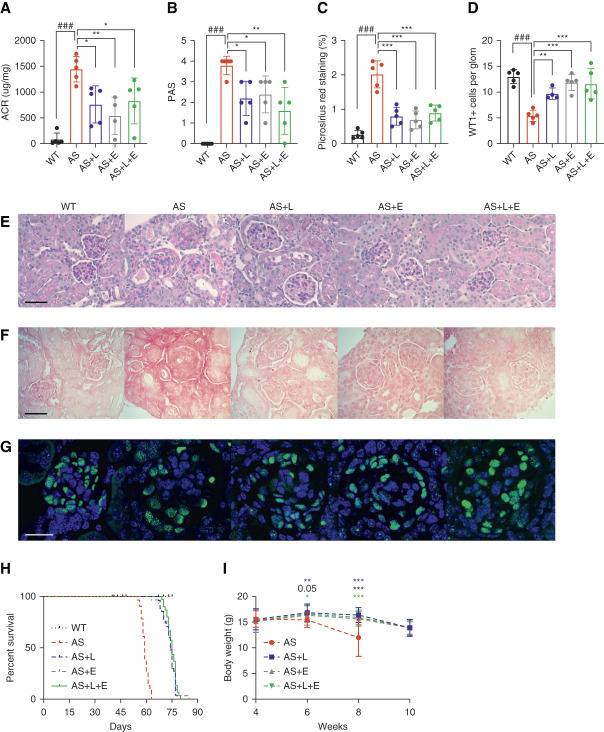
**Linagliptin improves renal function in a mouse model of AS**. (A) Urinary ACR ratio in WT and AS mice fed on a normal chow or normal chow complemented with linagliptin (L, 40 mg/kg), empagliflozin (E, 70 mg/kg), or the combination of linagliptin and empagliflozin (L+E). Urine samples were collected at the time of sacrifice (*n*=4–5). (B and E) Bar graph analysis and representative images of PAS staining of kidney cortex sections showing the mesangial expansion score (scale bar: 50 *μ*m; *n*=5). (C and F) Bar graph analysis and representative Picrosirius red staining showing the quantification of fibrosis in kidney cortex sections (scale bar: 50 *μ*m; *n*=4–5). (D and G) Bar graph quantification showing the average number of WT1-positive podocytes per glomerulus. Representative images of kidney cortex sections stained with WT1 (green) to detect podocytes and DAPI (blue) to reveal nuclei (scale bar: 25 *μ*m, *n*=4–5). (H) Survival curve (*n*=8, 28, 32, 34, 36) of AS mice on different treatment regiments versus normal diet starting at age 4 weeks, compared with age-matched WT control mice. (I) Body weight was measured biweekly in AS mice on different treatments (*n*=27, 27, 30, 30). *P < 0.05, **P < 0.01, ***P (###*P*) < 0.001. #AS compared with WT, two-tailed Student *t* test. *Treatment groups compared with AS, one-way ANOVA followed by Holm-Sidak multiple comparison. ACR, albumin-to-creatinine ratio; DAPI, 4’,6-diamidino-2-phenylindole WT1, Wilms tumor 1.

DPP4i have been used independently and along with SGLT2i as an add-on glucose lowering therapy.^33^ As the removal of glucose stimulates increased utilization of lipids as an energy source,^19^ DPP4i may have the potential to simultaneously reduce renal lipids in AS kidneys. To investigate the renal protective effect of DPP4i and compare their effect with SGLT2i, AS mice were treated with Lina, Empa, and the combination of Lina and Empa. At age 8 weeks, the mice were euthanized. Urine samples were collected, and the albumin-to-creatinine ratios were determined. We found that proteinuria was significantly reduced in AS mice treated with Lina, Empa, or Lina+Empa, but there was no significant difference in proteinuria levels between Lina-, Empa-, and Lina+Empa-treated AS mice (Figure 2A). In addition, reduced glomerulosclerosis and tubulointerstitial fibrosis were observed in AS mice treated with Lina, Empa, or Lina+Empa (Figure 2, B, C, E, and F). AS mice exhibited a significant reduction in the number of podocytes per glomerulus, as demonstrated by a decrease in the number of Wilms tumor 1–positive cells per glomerulus (Figure 2, D and G). All treatments prevented podocyte loss in AS mice. To investigate if reduced proteinuria in treated mice is associated with a prolonged life span of KO mice, a survival study was performed. Treatment of AS mice with Lina, Empa, or Lina+Empa significantly prolongs the survival of AS mice (Figure 2H). The median survival for untreated KO mice was 59 days, whereas KO mice on all treatment regiments survived approximately 14 days longer (median survival 74 days for AS+L and AS+E, 75 days for AS+L+E) (Figure 2H). Body weight was determined biweekly over a 10-week period. We found that treatment of AS mice with Lina, Empa, or Lina+Empa prevented the significant loss of body weight observed in untreated AS mice (Figure 2I). We performed MALDI-MSI to measure AdA, EPA, and glucose levels in the kidneys of AS mice fed a normal chow diet or a diet supplemented with Lina, Empa, or a combination of both. However, because of the large SD in the treatment groups, we were unable to detect any significant difference between the treatment groups and the control (Supplemental Table 2).

### Effects of Lina on Respiration Differ between AS Podocytes and Tubular Cells

**Figure 3:**
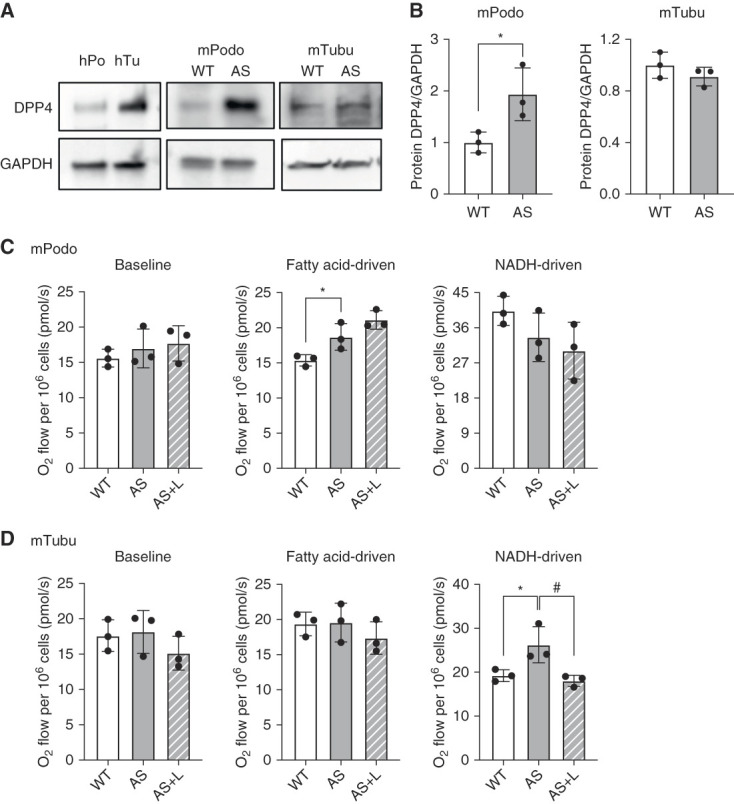
**Linagliptin reduces NADH-driven respiration in AS tubular cells**. (A) Western blot images showing DPP4 expression in cultured human podocytes (hPodo) and tubular cells (hTubu), as well as mouse podocytes (mPodo) and proximal tubular cells (mTubu) derived from WT and AS mice (*n*=3). (B) Quantification of DPP4 protein expression in WT and AS podocytes and tubular cells (*n*=3). (C and D) Bar graph analysis of baseline and substrate-driven OCRs in WT podocytes and tubular cells, as well as in (C) AS podocytes (D) and tubular cells treated with or without linagliptin (E) (*n*=3). *P and #*P* < 0.5. Two-tailed Student *t* test. DPP4, dipeptidyl peptidase-4; NADH, nicotinamide adenine dinucleotide OCR, oxygen consumption rate.

To identify the specific cell types that could potentially benefit from Lina treatment, we examined the expression of DPP4 in human podocytes and tubular cells. Our analysis confirmed DPP4 protein expression in both cell types (Figure 3A, full image: Supplemental Figure 2). We performed immunostaining for synaptopodin and DPP4 in the kidney cortex of WT and AS mice. Representative images reveal that DPP4 is present in both tubules and glomeruli, with some colocalization observed in podocytes (Supplemental Figure 3). To investigate the protective effects of Lina on renal cells in AS, we established immortalized mouse podocyte and tubular cell lines from WT mice and AS mice. We found significantly increased DPP4 protein expression in AS compared with WT podocytes (Figure 3, A and B), whereas DPP4 protein expression was similar in tubular cells derived from WT and AS mice (Figure 3, A and B).

We previously demonstrated that SGLT2i reduce lipotoxicity in AS podocytes by shifting their energy source from fatty acids to glucose for respiration, although AS podocytes have a similar baseline respiration rate as WT podocytes.^19^ Here, we have confirmed comparable results in the respiration assay between AS and WT podocytes and tubular cells (Figure 3, C and D). We attributed this observation to the fact of an increased removal of glucose by SGLT2i.^19^ In this study, we aimed to evaluate the effect of Lina, which lowers glucose levels by increasing insulin secretion, on substrate-driven respiration in podocytes and tubular cells derived from AS mice. We found that Lina did not alter the baseline or fatty acid-driven respiration in both AS podocytes and tubular cells (Figure 3, C and D). Interestingly, while Lina treatment had also no effect on in AS podocytes, we found that Lina treatment reduces glucose/nicotinamide adenine dinucleotide-driven respiration in AS tubular cells (Figure 3, C and D). These results suggest that Lina may have differential effects on substrate-driven respiration in podocytes and tubular cells derived from AS mice. Further investigations are warranted to elucidate the detailed underlying mechanisms and to explore the potential therapeutic implications of these observations.

## Discussion

With this study, we investigated the distribution of metabolites and altered metabolism in kidney tissues from AS and WT mice using the powerful MALDI-MSI technique. We demonstrate, for the first time, the dysregulation of certain metabolites in the kidneys of AS mice. Specifically, we found perturbations in the metabolism of AdA and EPA (Figure 1). These two PUFAs are derived from omega-6 and omega-3 fatty acids, respectively. Previous reports indicated increased AdA levels in the medium of podocytes depleted of decaprenyl diphosphate synthase subunit 2.^34^ Podocytes depleted of decaprenyl diphosphate synthase subunit 2 protein is responsible for coenzyme Q biosynthesis, which plays a critical role in the electron transport chain and ATP production.^35,36^ Thus, it seems possible that AdA is involved in energy generation. A high-fat diet triggers renal lipotoxicity, resulting in increased levels of renal AdA and its metabolites in mice, while reducing EPA and its metabolite levels.^37^ This metabolic imbalance was found to be improved by activating AMP-activated protein kinase.^37^ These findings are consistent with our observations in kidney sections of AS mice, which suggests that renal lipotoxicity of metabolic and nonmetabolic origin may share similar pathophysiological processes involved in PUFA metabolism.

We also found elevated levels of glucose and glucose-6 phosphate in the kidneys of AS mice (Figure 1). Glucose-6 phosphate serves as a critical intermediate in glucose metabolism.^38,39^ Although the reason for the accumulation of glucose and its metabolic intermediate in the kidneys of AS mice remains unclear, compounds used to treat type 2 diabetes have been tested in experimental models of AS. One such compound is metformin, an antidiabetic agent, which demonstrated renal protective effects in AS mice. These effects include reduced proteinuria, renal inflammation, fibrosis, and glomerular injury.^40^ The renal protective effect of metformin thereby seems to be associated with the improvement of metabolic imbalances in glycolysis, the tricarboxylic acid cycle, and lipid metabolism.^40^ Similarly, we demonstrate that Empa improves kidney function and lifespan in AS mice by shifting the preferred energy source from glucose to lipids leading to reduced renal lipotoxicity.^19^ These observations suggest that diabetic drugs, such as DPP4i, may be considered for the treatment of patients with nondiabetic CKD.

To assess the potential use of DPP4i in treating nondiabetic CKD, we next treated AS mice with Lina and compared disease progression to untreated and Empa-treated AS mice (Figure 2). The dosage of Lina used in mouse models varies. In research by Takahashi *et al.*,^41^ a regimen of 3 mg/kg per day Lina and/or 30 mg/kg per day Empa was used for treating diabetic db/db mice. This treatment led to a significant reduction in body weight, especially when mice were given Empa alone or in combination with Lina. In addition, in another study, ob/ob mice were treated with a higher dose of 100 mg/kg per day evogliptin, which alleviated cardiac lipotoxicity and prevented the development of diabetic cardiomyopathy.^42^ In our mouse model, we chose to feed the mice food containing 40 mg/kg Lina, with their daily food intake approximately 3 g, a dose slightly higher than that used in the study by Takahashi. This dosing was selected based on preliminary studies where an extension in lifespan was observed. Given these promising results, we have decided to continue using this dose in our study.

As anticipated, Lina treatment also improved albuminuria, glomerulosclerosis, fibrosis, podocyte loss, and bodyweight loss in the experimental AS model. The effects of Lina treatment were similar to those observed with Empa treatment, and the combination of both drugs did not further improve renal function in AS mice. The absence of an additive effect was surprising, as our *in vitro* mechanistic studies demonstrated that Empa and Lina affect similar metabolic pathways in different cell types in AS, that is podocytes versus tubular cells, respectively. It is, therefore, possible that other compensatory mechanisms occur *in vivo*. Although the survival of AS mice in all treatment groups was extended, mice still succumbed to the disease by age 10.5 weeks. Thus, additional studies to investigate whether other combination treatment strategies, such as the combination of Lina or Empa with inhibitors of the renin-angiotensin-aldosterone system^43^ or finerenone, may be superior and improve the survival of AS mice are warranted. Another limitation of our data is the substantial SD observed within the treatment groups. This variability has impeded our capacity to identify significant differences between the treatment groups and the control group. Therefore, further MALDI-MSI analysis to investigate the effects of Lina on glucose and lipid metabolism is warranted.

We also established immortalized mouse podocytes and tubular cells from WT and AS mice^14,19^ to investigate whether Lina modulates cell respiration. We measured the oxygen consumption rate using high-resolution respirometry (Figure 3). In previous research, we found no differences in endogenous respiration between WT and AS podocytes and tubular cells.^19^ However, AS tubular cells treated with Lina exhibited decreased glucose-driven respiration, whereas endogenous respiration and fatty acid-driven respiration remained unaffected. Interestingly, Lina treatment did not affect podocyte respiration, suggesting that tubular cells may be the primary target of Lina-induced metabolic changes. Because hyperglycemia and glycosuria are not symptoms of AS, it seems possible that the increased renal glucose content is the consequence of altered glucose transport within the kidney. Increased glucose content is also known to affect the expression of extracellular matrix proteins, leading to increased extracellular matrix deposition.^44,45^ Thus, we can speculate that the intrarenal accumulation of lipids, glucose, and their metabolites may cause glucolipotoxicity^46^ and that it is necessary to achieve a reduction in both lipid and glucose content in the kidney to prevent disease progression in AS. Our previous finding that Empa treatment improves kidney function in AS mice^19^ may be attributed to both increased lipid utilization and glucose excretion, thereby reducing glucolipotoxicity.

The evolving data from cardiovascular outcomes trials suggest a potential kidney-protective effect of GLP-1 receptor agonists in individuals with type 2 diabetes and CKD, despite the lack of explicit indications for kidney improvement.^47^ In cardiovascular outcomes trials, findings related to the kidneys have often been presented as secondary evaluations after the primary emphasis on cardiovascular results.^48^ GLP-1 receptor agonists exert indirect influence on typical risk factors for diabetic kidney disease, such as high blood sugar, hypertension, obesity, and dyslipidemia,^48^ while also show potential direct effects on the kidneys by reducing oxidative stress,^49^ inflammation,^50,51^ and natriuresis.^16^ Further research could illuminate their specific role in managing renal health for these patient populations.

In summary, we have identified several dysregulated metabolites in the kidney cortices of AS mice. Among them, we found distinct differences in the content and distribution of the two PUFAs, AdA and EPA, in AS compared with WT kidneys. Interestingly, we also found increased glucose levels in AS kidneys. We furthermore demonstrated that Lina can effectively reduce the decline in kidney function and improve the lifespan of AS mice and that Lina reduces glucose-induced respiration in AS tubular cells. Taken together, our studies suggest that inhibiting the generation of glucose metabolites could represent a potential therapeutic strategy for treating AS. Additional studies are necessary to investigate the detrimental effects of glucose accumulation in the kidney during AS progression and to determine whether reducing renal glucose content can be a viable treatment option for the treatment of patients with AS.

## Supplementary Material

## Data Availability

All data are included in the manuscript and/or supporting information.

## Supplemental Material

Supplemental Methods

Supplemental Figure 1. Alport syndrome (AS) and wild-type (WT) kidney metabolites distinctly cluster together in principal component (PC) analysis.

Supplemental Figure 2. Full western blot images with molecular weight markers.

Supplemental Figure 3. Immunohistochemistry staining of mouse kidney cortex for DPP4 and synaptopodin (scale bar: 35 *μ*m).

Supplemental Table 1. Abundance of key discriminant metabolites between Alport syndrome (AS) and wild-type (WT) kidneys. Student *t* test.

Supplemental Table 2. Mean ion intensity of the treatment group.

Supplemental References
