# Supplementary material for: Metabolic Analysis and Renal Protective Effects of Linagliptin and Empagliflozin in Alport Syndrome
Source: Kidney360. 2024 May 23;5(7):1002–11. doi: 10.34067/KID.0000000000000472 (PMC11296534; doi:10.34067/KID.0000000000000472)
Supplement: Supplementary file 1 [file kidney360-5-1002-s001.pdf]

ASN American Society of Nephrology | JASN Journal of the American Society of Nephrology | KIDNEY360 Accessing Our World From Every Angle | CJASN Clinical Journal of the American Society of Nephrology

ASN Journal Disclosure Form

As per ASN journal policy, I have disclosed any financial relationship or commitment held by myself and/or my spouse/partner in the past 36 months as included below. I have listed my Current Employer below to indicate there is a relationship requiring disclosure. If no relationship exists, my Current Employer is not listed.

F. Fontanesi reports the following:
Employer: University of Miami

I understand that the information above will be published within the journal article, if accepted, and that failure to comply and/or to accurately and completely report the potential financial conflicts of interest could lead to the following: 1) Prior to publication, article rejection, or 2) Post-publication, sanctions ranging from, but not limited to, issuing a correction, reporting the inaccurate information to the authors' institution, banning authors from submitting work to ASN journals for varying lengths of time, and/or retraction of the published work.

Name: Flavia Fontanesi
Manuscript ID: K360-2023-000660R1
Manuscript Title: Metabolic Analysis and Renal Protective Effects of Linagliptin and Empagliflozin in Alport Syndrome
Date of Completion: January 21, 2024
Disclosure Updated Date: January 21, 2024

ASN
American Society of Nephrology
JASN
Journal of the American Society of Nephrology
KIDNEY360
Accessing Our World From Every Angle
CJASN
Clinical Journal of the American Society of Nephrology

ASN Journal Disclosure Form

As per ASN journal policy, I have disclosed any financial relationship or commitment held by myself and/or my spouse/partner in the past 36 months as included below. I have listed my Current Employer below to indicate there is a relationship requiring disclosure. If no relationship exists, my Current Employer is not listed.

A. Fornoni reports the following:
Employer: University of Miami; Consultancy: Dimerix, Travere, Bayer, DinaQOR; Ownership Interest: I am CSO and Vice-President of L&F Health LLC.; I am shareholder for Zyversa Therapeutics; I am shareholder of River 3 Renal Corp; I am shareholder of UpToDate; Research Funding: Aurinia, Pfizer, Travere; Patents or Royalties: Patent on the use of cyclodextrin for the treatment of kidney diseases.; Patent on the use of small molecule inducers of cholesterol efflux.; I own shares in Zyversa Therapeutics and R3R.; and Other Interests or Relationships: I am inventor on 5 pending US patents and one published patent.

I understand that the information above will be published within the journal article, if accepted, and that failure to comply and/or to accurately and completely report the potential financial conflicts of interest could lead to the following: 1) Prior to publication, article rejection, or 2) Post-publication, sanctions ranging from, but not limited to, issuing a correction, reporting the inaccurate information to the authors' institution, banning authors from submitting work to ASN journals for varying lengths of time, and/or retraction of the published work.

Name: Alessia Fornoni
Manuscript ID: K360-2023-000660R1
Manuscript Title: Metabolic Analysis and Renal Protective Effects of Linagliptin and Empagliflozin in Alport Syndrome
Date of Completion: January 22, 2024
Disclosure Updated Date: September 5, 2023

ASN American Society of Nephrology | JASN Journal of the American Society of Nephrology | KIDNEY360 Accessing Our World From Every Angle | CJASN Clinical Journal of the American Society of Nephrology

ASN Journal Disclosure Form

As per ASN journal policy, I have disclosed any financial relationship or commitment held by myself and/or my spouse/partner in the past 36 months as included below. I have listed my Current Employer below to indicate there is a relationship requiring disclosure. If no relationship exists, my Current Employer is not listed.

M. Ge has nothing to disclose.

I understand that the information above will be published within the journal article, if accepted, and that failure to comply and/or to accurately and completely report the potential financial conflicts of interest could lead to the following: 1) Prior to publication, article rejection, or 2) Post-publication, sanctions ranging from, but not limited to, issuing a correction, reporting the inaccurate information to the authors' institution, banning authors from submitting work to ASN journals for varying lengths of time, and/or retraction of the published work.

Name: Mengyuan Ge
Manuscript ID: K360-2023-000660R1
Manuscript Title: Metabolic Analysis and Renal Protective Effects of Linagliptin and Empagliflozin in Alport Syndrome
Date of Completion: March 6, 2024
Disclosure Updated Date: March 6, 2024

ASN
American Society of Nephrology
JASN
Journal of the American Society of Nephrology
KIDNEY360
Accessing Our World From Every Angle
CJASN
Clinical Journal of the American Society of Nephrology

ASN Journal Disclosure Form

As per ASN journal policy, I have disclosed any financial relationship or commitment held by myself and/or my spouse/partner in the past 36 months as included below. I have listed my Current Employer below to indicate there is a relationship requiring disclosure. If no relationship exists, my Current Employer is not listed.

J. Kim reports the following:
Research Funding: NIDDK K01

I understand that the information above will be published within the journal article, if accepted, and that failure to comply and/or to accurately and completely report the potential financial conflicts of interest could lead to the following: 1) Prior to publication, article rejection, or 2) Post-publication, sanctions ranging from, but not limited to, issuing a correction, reporting the inaccurate information to the authors' institution, banning authors from submitting work to ASN journals for varying lengths of time, and/or retraction of the published work.

Name: Jin Ju Kim
Manuscript ID: K360-2023-000660R1
Manuscript Title: Metabolic Analysis and Renal Protective Effects of Linagliptin and Empagliflozin in Alport Syndrome
Date of Completion: March 5, 2024
Disclosure Updated Date: May 22, 2023

ASN Journal Disclosure Form

As per ASN journal policy, I have disclosed any financial relationship or commitment held by myself and/or my spouse/partner in the past 36 months as included below. I have listed my Current Employer below to indicate there is a relationship requiring disclosure. If no relationship exists, my Current Employer is not listed.

S. Merscher reports the following:
Employer: University of Miami; Ownership Interest: Zyversa Therapeutics, Inc.; Research Funding: Aurinia Pharmaceuticals; Pfizer; Patents or Royalties: I am Co-inventor on several pending and issued patents. I have an indirect equity interest in, and potential patent licensing income from, ZyVersa Therapeutics, Inc. by virtue of assignment and licensure of a patent estate.; and Advisory or Leadership Role: Review Editor for Frontiers in Medicine, section nephrology; Associate Editor for Frontiers in Medicine, section Nephrology.

I understand that the information above will be published within the journal article, if accepted, and that failure to comply and/or to accurately and completely report the potential financial conflicts of interest could lead to the following: 1) Prior to publication, article rejection, or 2) Post-publication, sanctions ranging from, but not limited to, issuing a correction, reporting the inaccurate information to the authors' institution, banning authors from submitting work to ASN journals for varying lengths of time, and/or retraction of the published work.

Name: Sandra M. Merscher
Manuscript ID: K360-2023-000660R1
Manuscript Title: Metabolic Analysis and Renal Protective Effects of Linagliptin and Empagliflozin in Alport Syndrome
Date of Completion: March 5, 2024
Disclosure Updated Date: March 5, 2024

ASN American Society of Nephrology JASN Journal of the American Society of Nephrology KIDNEY360 Accessing Our World From Every Angle CJASN Clinical Journal of the American Society of Nephrology

ASN Journal Disclosure Form

As per ASN journal policy, I have disclosed any financial relationship or commitment held by myself and/or my spouse/partner in the past 36 months as included below. I have listed my Current Employer below to indicate there is a relationship requiring disclosure. If no relationship exists, my Current Employer is not listed.

J. Molina David has nothing to disclose.

I understand that the information above will be published within the journal article, if accepted, and that failure to comply and/or to accurately and completely report the potential financial conflicts of interest could lead to the following: 1) Prior to publication, article rejection, or 2) Post-publication, sanctions ranging from, but not limited to, issuing a correction, reporting the inaccurate information to the authors' institution, banning authors from submitting work to ASN journals for varying lengths of time, and/or retraction of the published work.

Name: Judith T. Molina David
Manuscript ID: K360-2023-000660R1
Manuscript Title: Metabolic Analysis and Renal Protective Effects of Linagliptin and Empagliflozin in Alport Syndrome
Date of Completion: March 19, 2024
Disclosure Updated Date: March 19, 2024

ASN American Society of Nephrology | JASN Journal of the American Society of Nephrology | KIDNEY360 Accessing Our World From Every Angle | CJASN Clinical Journal of the American Society of Nephrology

ASN Journal Disclosure Form

As per ASN journal policy, I have disclosed any financial relationship or commitment held by myself and/or my spouse/partner in the past 36 months as included below. I have listed my Current Employer below to indicate there is a relationship requiring disclosure. If no relationship exists, my Current Employer is not listed.

R. Njeim has nothing to disclose.

I understand that the information above will be published within the journal article, if accepted, and that failure to comply and/or to accurately and completely report the potential financial conflicts of interest could lead to the following: 1) Prior to publication, article rejection, or 2) Post-publication, sanctions ranging from, but not limited to, issuing a correction, reporting the inaccurate information to the authors' institution, banning authors from submitting work to ASN journals for varying lengths of time, and/or retraction of the published work.

Name: Rachel Njeim
Manuscript ID: K360-2023-000660R1
Manuscript Title: Metabolic Analysis and Renal Protective Effects of Linagliptin and Empagliflozin in Alport Syndrome
Date of Completion: January 21, 2024
Disclosure Updated Date: January 21, 2024

ASN American Society of Nephrology | JASN Journal of the American Society of Nephrology | KIDNEY360 Accessing Our World From Every Angle | CJASN Clinical Journal of the American Society of Nephrology

ASN Journal Disclosure Form

As per ASN journal policy, I have disclosed any financial relationship or commitment held by myself and/or my spouse/partner in the past 36 months as included below. I have listed my Current Employer below to indicate there is a relationship requiring disclosure. If no relationship exists, my Current Employer is not listed.

M. Pieper reports the following:
Employer: Boehringer Ingelheim Pharma GmbH & Co. KG; Research Funding: Boehringer Ingelheim Pharma GmbH & Co. KG; and Patents or Royalties: Boehringer Ingelheim Pharma GmbH & Co. KG.

I understand that the information above will be published within the journal article, if accepted, and that failure to comply and/or to accurately and completely report the potential financial conflicts of interest could lead to the following: 1) Prior to publication, article rejection, or 2) Post-publication, sanctions ranging from, but not limited to, issuing a correction, reporting the inaccurate information to the authors' institution, banning authors from submitting work to ASN journals for varying lengths of time, and/or retraction of the published work.

Name: Michael P. Pieper
Manuscript ID: K360-2023-000660R1
Manuscript Title: Metabolic Analysis and Renal Protective Effects of Linagliptin and Empagliflozin in Alport Syndrome
Date of Completion: April 2, 2024
Disclosure Updated Date: April 2, 2024

ASN American Society of Nephrology
JASN Journal of the American Society of Nephrology
KIDNEY360 Accessing Our World From Every Angle
CJASN Clinical Journal of the American Society of Nephrology

ASN Journal Disclosure Form

As per ASN journal policy, I have disclosed any financial relationship or commitment held by myself and/or my spouse/partner in the past 36 months as included below. I have listed my Current Employer below to indicate there is a relationship requiring disclosure. If no relationship exists, my Current Employer is not listed.

K. Sharma reports the following:
Employer: University of Texas Health San Antonio; South Texas VA HealthCare System; Consultancy: Otsuka, Bayer, Cara Therapeutics; Ownership Interest: SygnaMap; Research Funding: Boehringer Ingelheim; Honoraria: Pfizer; Patents or Royalties: University of Texas Health San Antonio; Advisory or Leadership Role: DSMB for Cara Therapeutics. Founder, SygnaMap. Reata; and Other Interests or Relationships: Texas Renal Coalition, ex-board member of Texas Kidney Foundation.

I understand that the information above will be published within the journal article, if accepted, and that failure to comply and/or to accurately and completely report the potential financial conflicts of interest could lead to the following: 1) Prior to publication, article rejection, or 2) Post-publication, sanctions ranging from, but not limited to, issuing a correction, reporting the inaccurate information to the authors' institution, banning authors from submitting work to ASN journals for varying lengths of time, and/or retraction of the published work.

Name: Kumar Sharma
Manuscript ID: K360-2023-000660R1
Manuscript Title: Metabolic Analysis and Renal Protective Effects of Linagliptin and Empagliflozin in Alport Syndrome
Date of Completion: April 1, 2024
Disclosure Updated Date: March 27, 2024

ASN American Society of Nephrology
JASN Journal of the American Society of Nephrology
KIDNEY360 Accessing Our World From Every Angle
CJASN Clinical Journal of the American Society of Nephrology

ASN Journal Disclosure Form

As per ASN journal policy, I have disclosed any financial relationship or commitment held by myself and/or my spouse/partner in the past 36 months as included below. I have listed my Current Employer below to indicate there is a relationship requiring disclosure. If no relationship exists, my Current Employer is not listed.

I. Tamayo has nothing to disclose.

I understand that the information above will be published within the journal article, if accepted, and that failure to comply and/or to accurately and completely report the potential financial conflicts of interest could lead to the following: 1) Prior to publication, article rejection, or 2) Post-publication, sanctions ranging from, but not limited to, issuing a correction, reporting the inaccurate information to the authors' institution, banning authors from submitting work to ASN journals for varying lengths of time, and/or retraction of the published work.

Name: Ian M. Tamayo
Manuscript ID: K360-2023-000660R1
Manuscript Title: Metabolic Analysis and Renal Protective Effects of Linagliptin and Empagliflozin in Alport Syndrome
Date of Completion: March 26, 2024
Disclosure Updated Date: May 19, 2023

ASN American Society of Nephrology JASN Journal of the American Society of Nephrology KIDNEY360 Accessing Our World From Every Angle CJASN Clinical Journal of the American Society of Nephrology

ASN Journal Disclosure Form

As per ASN journal policy, I have disclosed any financial relationship or commitment held by myself and/or my spouse/partner in the past 36 months as included below. I have listed my Current Employer below to indicate there is a relationship requiring disclosure. If no relationship exists, my Current Employer is not listed.

G. Zhang has nothing to disclose.

I understand that the information above will be published within the journal article, if accepted, and that failure to comply and/or to accurately and completely report the potential financial conflicts of interest could lead to the following: 1) Prior to publication, article rejection, or 2) Post-publication, sanctions ranging from, but not limited to, issuing a correction, reporting the inaccurate information to the authors' institution, banning authors from submitting work to ASN journals for varying lengths of time, and/or retraction of the published work.

Name: Guanshi Zhang
Manuscript ID: K360-2023-000660R1
Manuscript Title: Metabolic Analysis and Renal Protective Effects of Linagliptin and Empagliflozin in Alport Syndrome
Date of Completion: March 4, 2024
Disclosure Updated Date: March 4, 2024
