# Supplementary material for: Metabolic Analysis and Renal Protective Effects of Linagliptin and Empagliflozin in Alport Syndrome
Source: Kidney360. 2024 May 23;5(7):1002–11. doi: 10.34067/KID.0000000000000472 (PMC11296534; doi:10.34067/KID.0000000000000472)
Supplement: Supplementary file 2 [file kidney360-5-1002-s002.pdf]

Table of Contents for the Supplemental Material

Supplemental Methods

Supplemental Figure 1

Supplemental Figure 2

Supplemental Figure 3

Supplemental Table 1

Supplemental Table 2

Reference

Supplemental Methods

Urinary albumin-to-creatinine ratios

Morning spot urine samples were collected bi-weekly. Urinary albumin-to-creatinine ratios were determined using the Mouse Albumin ELISA Kit (Bethyl Laboratories) and Creatinine LiquiColor (Stanbio). The albuminuria values are expressed as µg of albumin per mg of creatinine.

Kidney histology analysis

Perfused kidneys were fixed in 10% formalin, paraffin-embedded, and cut into 4 µm thick sections. To investigate mesangial expansion, periodic acid-Schiff (PAS) staining was conducted using a standard protocol. Mesangial expansion was visualized under a light microscope (Olympus BX41, Tokyo, Japan), and semi-quantitative analysis (scored on a scale of 0-5) was performed in a blinded manner, examining 10 glomeruli per section.

To measure fibrosis, Picrosirius Red staining was performed. Paraffin-embedded sections were deparaffinized using xylene and a graded alcohol series. The sections were then stained with Picrosirius Red in saturated aqueous picric acid for 1 hour. After staining, the sections were examined under a light microscope (Olympus BX41, Tokyo, Japan), followed by analysis using Fiji/Image J.

Immunofluorescence staining

To measure podocyte number per glomerulus, glomerular sections embedded in OCT were stained with a Wilms tumor 1 (WT1) antibody (Santa Cruz Biotechnology, Dallas, TX, sc-192, 1:300), followed by a secondary antibody (Invitrogen, Waltham, MA, A-11008, 1:500) and Mounting Medium with DAPI (Vectorlabs, Newark, CA, H-1200). To study the localization of DPP4, kidney cortex sections were stained with Synaptopodin (Santa Cruz Biotechnology, Dallas, TX, sc-515842, 1:100) and DPP4 (Cell Signaling Technology, Danvers, MA, 67138, 1:100) with secondary antibodies (Invitrogen, Waltham, MA, A-11001 & A-21244, 1:500). Images were acquired using Olympus IX81 confocal microscope (Tokyo, Japan) coupled with a 60 x oil immersion objective lens and images were processed using Fiji/Image J.

Cell culture

The establishment of conditionally immortalized mouse podocyte and tubular cell lines has been previously described[1,2]. In brief, the immortalized cell lines were cultured in RPMI growth medium containing 10% FBS, 1% penicillin/streptomycin, and 100 U/ml IFNγ at 33 °C under permissive conditions. Podocyte cell lines were shifted to non-permissive conditions at 37 °C without IFNγ for 12 days. For the experiments, the cultured cells were incubated with 50 nM linagliptin (Selleckchem, Houston, TX) or dimethylsulfoxide in the growth medium for 48 hours.

Western blot analysis

Cell lysates were prepared using a 3-[(3-cholamidopropyl)dimethylammonio]-1-propanesulfonic (CHAPS) acid buffer. The protein concentration was determined using the bicinchoninic acid (BCA) reagent from Thermo Scientific. Subsequently, 20 µg of protein extract was loaded onto SDS-polyacrylamide gel electrophoresis (SDS-PAGE) gels (Bio-Rad) with a concentration range of 4 to 20%. The gel transfer was conducted using the Trans-Blot Turbo Transfer System (Bio-Rad). For Western blot analysis, a standard protocol was followed, utilizing primary antibodies DPP4 (Cell Signaling Technology, #67138, 1:1,000) and GAPDH (Sigma-Aldrich, CB1001, 1:10,000), along with secondary antibodies including anti-mouse IgG horseradish peroxidase (HRP) (Promega, W402B, 1:10,000) and anti-rabbit IgG HRP (Promega, W401B, 1:10,000). The signal was detected using Radiance ECL (Azure, Dublin, CA) and captured using the Azure c600 Imaging System.

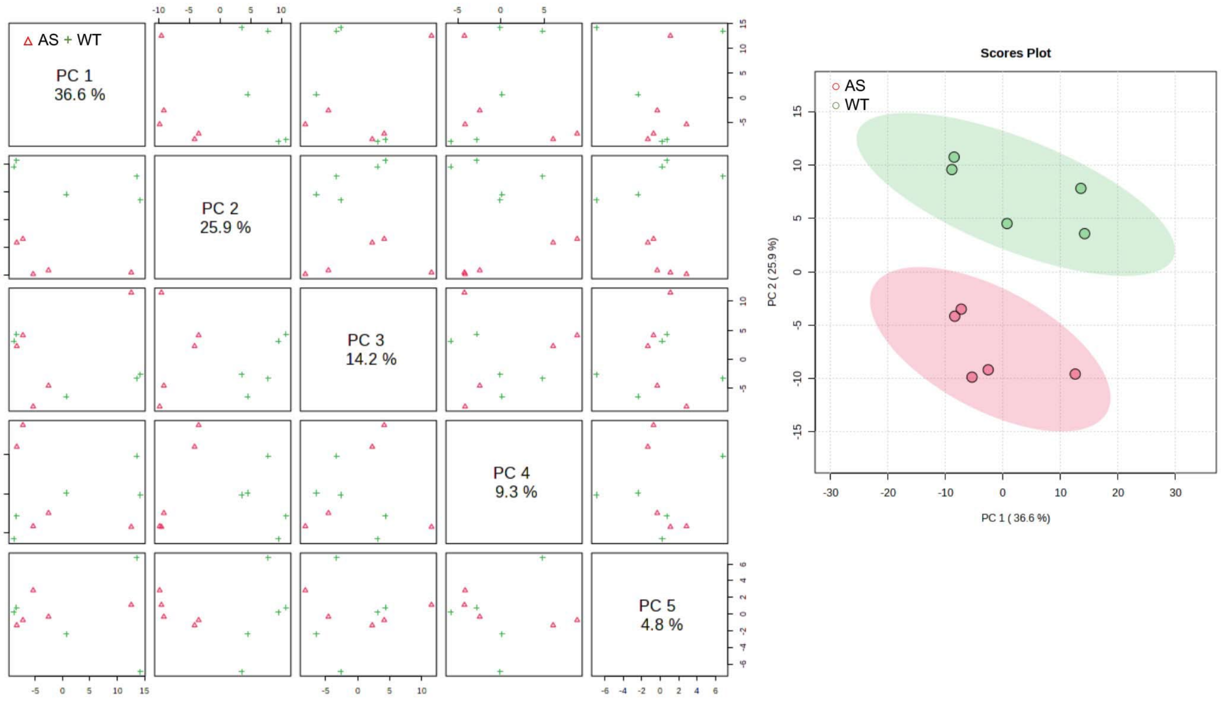

Supplemental Figure 1. Alport (AS) and wildtype (WT) kidney metabolites distinctly cluster together in principal component (PC) analysis.

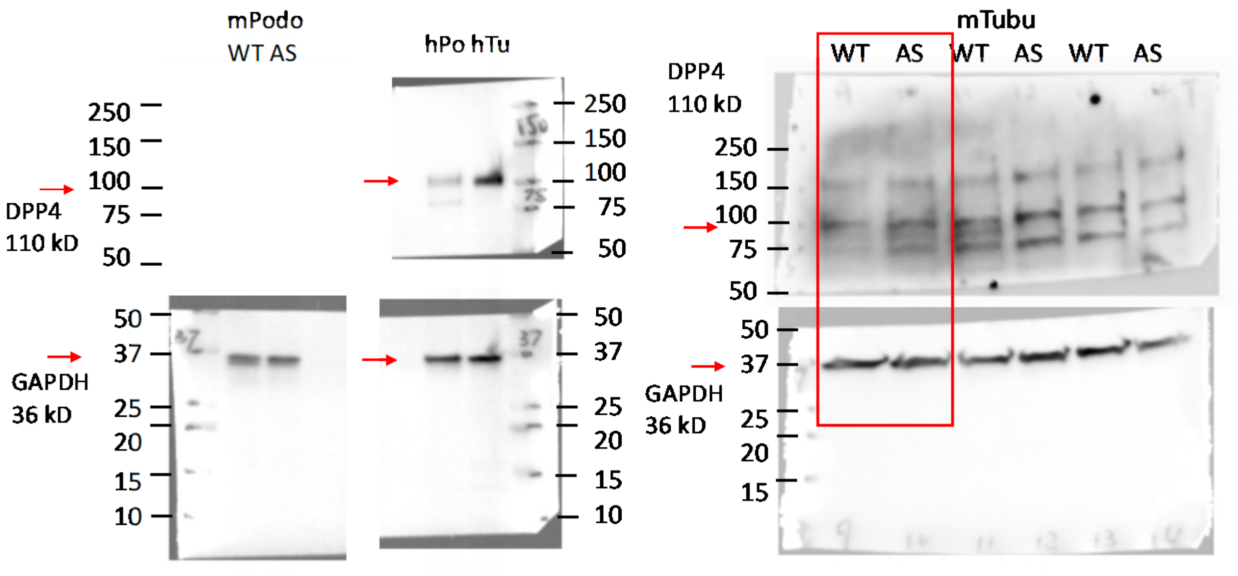

Supplemental Figure 2. Full Western Blot images with molecular weight markers.

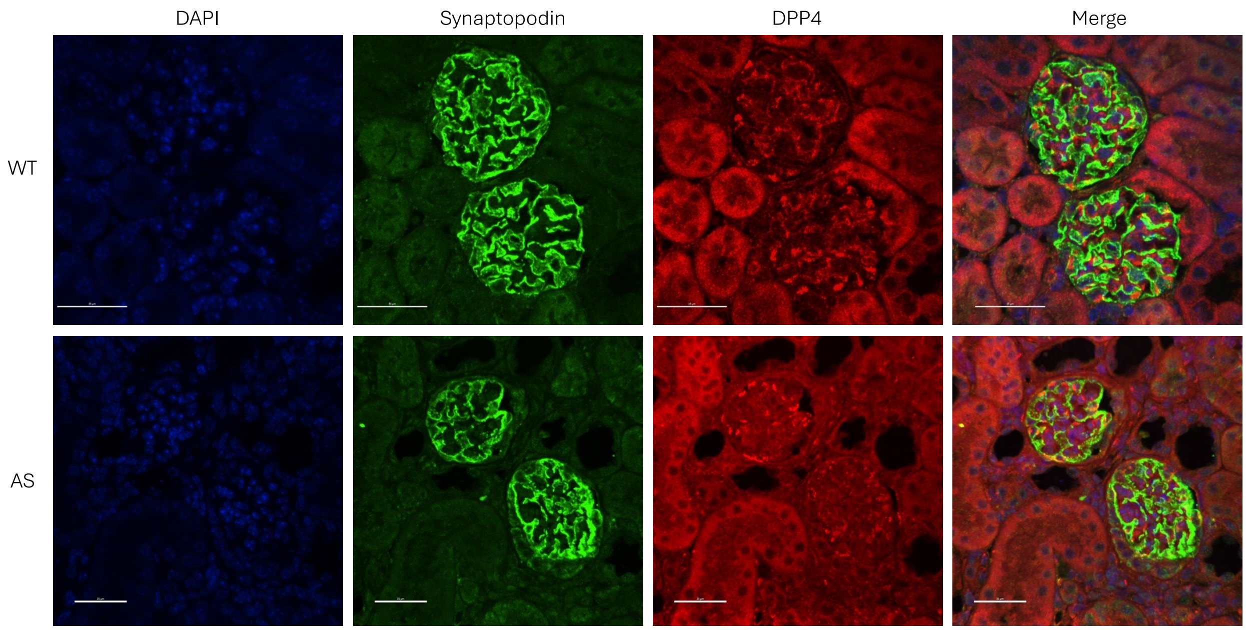

Supplemental Figure 3. Immunohistochemistry staining of mouse kidney cortex for DPP4 and Synaptopodin (scale bar: 35 µm).

Supplemental Table 1. Abundance of key discriminant metabolites between Alport (AS) and wildtype (WT) kidneys. Student's t-test.

Molecule ID	Ion m/z	AS Average	WT Average
Adrenic Acid	331.2642	1118.013456	406.2930215
Eicosapentaenoic Acid	301.2172	163.229433	211.6954267
Glucose	179.056	2723.955544	1075.3206
Citrulline	174.0883	107.1515556	0.316354536
Glycerophosphoethanolamine, PE(P-18:0/22:4)	778.5755	101.5468642	31.93592818
Phosphatidylglycerol, PG(22:6/22:6)	865.5024	57.85675239	2.354584286
Glutamine	145.0618	1211.21242	558.2280067
Lysophosphatidylcholine, lysoPE(18:0)	480.3095	1674.114453	2563.706143
Lysophosphatidic acid, lysoPA(16:0)	409.236	695.8214084	1123.194421
Phosphoinositol, PI(40:6)	909.5498	135.229226	403.1758867
Phenylacetylglycine	192.0665	270.475026	1.105155496
Phosphoserine, PS(40:7)	832.5133	64.96968096	119.1457542
Tetracosanoic Acid	367.3581	8.767387995	2.397266403
Glucose-6-Phosphate	259.0224	3012.327431	3729.160625
Lysophosphoethanolamine, lysoPE(18:1)	478.2938	261.6966881	452.4192478
Citric Acid	191.0196	1740.128829	235.368345
Eicosenoic Acid	309.2798	175.0464396	85.78896464
Uridine	243.0622	281.6797974	23.74957194
PE (3:0/18:4)	528.2731	2984.677811	1990.755871

Supplemental Table 2. Mean ion intensity of the treatment group.

	AS	AS+L	AS+E	AS+L+E
Adrenic Acid	1118±212	1562±299.4	1345±253	952.5±398.8
Eicosapentaenoic Acid	163.2±6.05	145.8±27.53	152.8±55.06	110.3±47.4
Glucose	2724±461.8	3546±1088	3640±1223	3893±241.9

Values are presented as Mean ± SD.

Reference

1. Ge M, Molina J, Kim JJ, et al. Empagliflozin reduces podocyte lipotoxicity in experimental Alport syndrome. Elife. May 2 2023;12. doi: 10.7554/eLife.83353.
2. Kim J-J, David JM, Wilbon SS, et al. Discoidin domain receptor 1 activation links extracellular matrix to podocyte lipotoxicity in Alport syndrome. EBioMedicine. 2021/01/01/ 2021;63:103162. doi: https://doi.org/10.1016/j.ebiom.2020.103162.
